# Interactive Blood Vessel Segmentation from Retinal Fundus Image Based on Canny Edge Detector

**DOI:** 10.3390/s21196380

**Published:** 2021-09-24

**Authors:** Alexander Ze Hwan Ooi, Zunaina Embong, Aini Ismafairus Abd Hamid, Rafidah Zainon, Shir Li Wang, Theam Foo Ng, Rostam Affendi Hamzah, Soo Siang Teoh, Haidi Ibrahim

**Affiliations:** 1 School of Electrical & Electronic Engineering, Engineering Campus, Universiti Sains Malaysia, Nibong Tebal 14300, Pulau Pinang, Malaysia; alexanderooi@student.usm.my (A.Z.H.O.); eeteoh@usm.my (S.S.T.); 2 Department of Ophthalmology, School of Medical Sciences, Health Campus, Universiti Sains Malaysia, Kubang Kerian 16150, Kelantan, Malaysia; zunaina@usm.my; 3 Department of Neurosciences, School of Medical Sciences, Health Campus, Universiti Sains Malaysia, Kubang Kerian 16150, Kelantan, Malaysia; aini_ismafairus@usm.my; 4 Brain and Behaviour Cluster, School of Medical Sciences, Health Campus, Universiti Sains Malaysia, Kubang Kerian 16150, Kelantan, Malaysia; 5 Oncological and Radiological Sciences Cluster, Advanced Medical and Dental Institute (AMDI), Universiti Sains Malaysia, SAINS@BERTAM, Kepala Batas 13200, Pulau Pinang, Malaysia; rafidahzainon@usm.my; 6 Faculty of Art, Computing and Creative Industry, Universiti Pendidikan Sultan Idris, Tanjong Malim 35900, Perak, Malaysia; shirli_wang@fskik.upsi.edu.my; 7 Centre of Global Sustainability Studies (CGSS), Level 5, Hamzah Sendut Library, Universiti Sains Malaysia, USM, Minden 11800, Pulau Pinang, Malaysia; tfng@usm.my; 8 Fakulti Teknologi Kejuruteraan Elektrik dan Elektronik, Universiti Teknikal Malaysia Melaka, Durian Tunggal 76100, Melaka, Malaysia; rostamaffendi@utem.edu.my

**Keywords:** blood vessels, edge segmentation, fundus images, retinal

## Abstract

Optometrists, ophthalmologists, orthoptists, and other trained medical professionals use fundus photography to monitor the progression of certain eye conditions or diseases. Segmentation of the vessel tree is an essential process of retinal analysis. In this paper, an interactive blood vessel segmentation from retinal fundus image based on Canny edge detection is proposed. Semi-automated segmentation of specific vessels can be done by simply moving the cursor across a particular vessel. The pre-processing stage includes the green color channel extraction, applying Contrast Limited Adaptive Histogram Equalization (CLAHE), and retinal outline removal. After that, the edge detection techniques, which are based on the Canny algorithm, will be applied. The vessels will be selected interactively on the developed graphical user interface (GUI). The program will draw out the vessel edges. After that, those vessel edges will be segmented to bring focus on its details or detect the abnormal vessel. This proposed approach is useful because different edge detection parameter settings can be applied to the same image to highlight particular vessels for analysis or presentation.

## 1. Introduction

Fundus photography (i.e., photography of the eye rear) is described as a method of reflecting light to get a photo of the half-transparent retinal tissues put on the imaging plane. Hence, fundus photos can be done by taking a series of photographs of the interior of the eye through the pupil. A fundus camera or retinal camera consists of a specialized intricate microscope used with a flash-enable‘d camera designed to photograph the interior surface of the eye. Recent developments even allow fundus photography to be done by smartphone with a conventional handheld indirect ophthalmoscopy lens [1,2].

There are multiple methods to perform fundus imaging, such as doing it with color filters [3], red-free filter [4,5], and angiography [5] such as sodium fluorescein, and indocyanine green [6]. The red-free filter is mainly used for detecting superficial lesions and some vascular abnormalities within the retina and surrounding tissue. Sodium fluorescein angiography is usually used to detect Cystoid Macular Oedema and Diabetic Retinopathy. Indocyanine green is used for detecting deeper choroidal diseases.

Many medical specialists such as optometrists, ophthalmologists, and orthoptists check for conditions or medical issues using fundus images of the retina [7]. Many eye diseases and systemic diseases can be revealed through the retina [8]. Besides that, fundus photographs can document how certain medical disorders changes or cause anomalies in the retina. Another function of fundus imaging is to monitor the retinal for medical disorders, like diabetes, age-macular degeneration, and neoplasm of the cranial nerves, eyeball, retinal or choroid [9]. Moreover, fundus imaging check-ups are required for people with diabetes mellitus once or twice each year [10], because diabetes can cause blindness which can be avoided by laser treatment if the diabetic retinopathy is detected early [10]. Moreover, monitoring the retina changes in fundus images is a way of checking people going through anti-malarial therapy [11].

Fundus imaging can help in emergency cases with people having sudden blindness, consistent headaches, or high diastolic pressure (≥120 mmHg) [12]. Fundus imaging can be used to detect papilledema and swollen optic discs signifying increased intracranial pressure, which are caused by medical issues such as hydrocephalus, benign intracranial hypertension (a.k.a. pseudotumor cerebri), and brain tumors [13]. Fundus imaging can also be used to detect optic nerve cupping, which indicates glaucoma damage due to the pressure in the eye being increased, and/or a loss of blood flow to the optic nerve that kills the nerve [14]. This causes the cup to become larger in comparison to the optic disc since the support structure is not there, leading to optic nerve cupping [15]. Fundus imaging can also detect pulmonary arterial hypertension (PAH), which is shown by arteriolar constriction, arteriovenous nicking, vascular wall changes, flame-shaped hemorrhages, cotton-wool spots, yellow hard exudates, and optic disk edema [16].

Retinal vessel segmentation is an important technique to inspect arteries and veins in the retina [17]. Many medical specialties use fundus imaging to identify many types of medical issues, and blood vessel segmentation is crucial in such an analysis process [7]. Over the years, many fundus vessel segmentation techniques have been created due to their importance [18]. The output from a manual segmentation can be considered as the correct output. Manual segmentation is simple, but it is a tedious process. It is also prone to errors [19] due to tiredness. Furthermore, there may be some differences between segmentation results from different persons because each person may translate the image differently. Hence, an easier and faster segmentation method should be designed.

Countless methods have been developed for edge detection used in image segmentation. Canny’s approach is an excellent edge detection method and can be implemented in many ways [20,21]. Yet, it is not perfect and still contains multiple flaws [22]. The amount of parameters means that there is an almost infinite amount of tweaks that can be made, only for a minor improvement in the results [23]. Besides that, the Gaussian smoothing used in Canny’s algorithm can cause the location of the edges to be off, depending on the size of the Gaussian kernel used [24]. There are multiple semi-automatic edge segmentation methods available such as Livewire [25] and Snakes [26]. However, these methods are for general images and not specialized for fundus images. Hence, a semi-automated approach should be further developed by modifying Canny’s edge detection method specialized for fundus images. Canny’s edge detection method is chosen because its parameters that require tweaking make it suitable to be tweaked using a graphical user interface (GUI) toolbar to save time.

Highlighting certain vessels can also be a useful tool for bringing focus to them during medical presentations or discussions [27]. The automated or semi-automated edge-detection function will save time as compared to manual segmentation in vessel segmentation [28]. Moreover, only the blood vessels of interest will be segmented for analysis, only allowing the necessary parts to be focused.

This paper proposes the implementation of semi-automated image segmentation in fundus imaging using a GUI-based implementation that allows applying different edge detection parameters on different areas of the same image. The implemented image segmentation technique will also be improved by adding new features to the Canny edge detection technique. Highlighting certain vessels can also be a useful tool to bring focus to it during medical presentations or discussions. This paper is organized into five sections. Section 2 will give some related preliminary information. Section 3 will explain our proposed interactive segmentation approach. Section 4 will present the results and discussions. Section 5 will give the conclusion from this study.

## 2. Preliminaries

This section is divided into three subsections. First, Section 2.1 will introduce the Canny edge detector, which is the base of our segmentation approach. Then, Section 2.2 will introduce related image processing techniques. Next, Section 2.3 will present Pratt’s Figure of Merit (PFOM) as the quantitative evaluation method.

### 2.1. Canny Edge Detection Technique

One of the standard edge detection techniques is the Canny edge detection technique [29]. The following are the algorithmic steps for the Canny edge detector:

1. As the edges would be affected by noise, the noise reduction technique is applied. For this purpose, the input image is convolved with a Gaussian filter.
2. Information about the edges—the edge magnitude and direction—is calculated from the gradient components. The gradient components are obtained from the first difference gradient operator.
3. Edge candidates are identified by applying the non-maximal or critical suppression to the gradient magnitude.
4. The edges are further refined by applying the threshold to the non-maximal suppression image.

Chang et al. [30] demonstrated a modified Canny edge detector for detecting retinal blood vessels, particularly small vessels. The edge detector is programmed as a local hysteresis thresholding value generator. This approach uses information of major vessel locations to specify a small neighborhood and generates local hysteresis threshold values to detect meaningful edges, which may be missed if we use the Canny edge detection alone. Thus, this method is able to detect edges of small blood vessels.

Yitao et al. [31] proposed and implemented an improved Cany edge detector algorithm to detect thin edges in fundus images. First, they modified the non-linear diffusion filter so that it can reduce the noise effectively while preserving the edges in the image. Then, to further reduce the impacts from noise, they consider the gradient magnitude from diagonal directions. Finally, they determine the double thresholds for the edge detection, which can be self-adaptively calculated from the average interclass variance. The experimental results show that their proposed method is robust and performs better than the classical Canny algorithm in terms of accuracy and precision.

### 2.2. Image Processing Techniques

When performing fundus imaging analysis, many researchers opt to use the green channel of the fundus image. This is because the retinal components are more intense in the fundus image’s green channel [32]. Furthermore, the green channel has the highest local contrast as compared to the red and blue channels. The intensity difference between blood vessels, exudates, and hemorrhages is best observed in the green channel, which is neither under-lit nor over-saturated [33].

One of the approaches to enhancing the image contrast is by using adaptive histogram equalization (AHE). AHE was first developed by Ketcham et al. [34] as a contrast enhancement method for aircraft cockpits. AHE changes each pixel value by using a transformation function that is derived locally, based on a neighborhood region. Thus, the enhancement adapts the information from the local image content. Yet, the ordinary AHE usually unnaturally amplifies the contrast in the near-constant areas of the image and increases the noise level in these regions. As a result, Pizer et al. [35] formulated a variant of AHE known as contrast limited AHE (CLAHE), which is able to reduce AHE’s noise amplification problem.

CLAHE has been utilized by many researchers as a processing technique to increase the contrast in fundus images. For example, dos Santos et al. [36] have used CLAHE to improve the contrast of fundus images before the images are fed into a neural network to detect the blood vessels. In the work of retinal-vessel segmentation by Aurangzeb et al. [37], the researchers have improved the parameter tuning in CLAHE by using Modified Particle Swarm Optimization (MPSO), which is able to find the optimized clip limit and the size of the contextual region. This approach has successfully increased the performance of both supervised and unsupervised machine learning models for this type of segmentation. On the other hand, Alwazzan et al. [38] has suggested a contrast enhancement method for color retinal fundus images. They pre-processed the green channel of the fundus image by a Wiener filter before passing it to the CLAHE algorithm. The enhanced green channel is then combined with the original red and blue channels to obtain the output image. Chang et al. [39] enhance the contrast of the green channel of the retinal image in their proposed blood vessel segmentation method. The green channel has also been utilized by Chatterjee et al. [40] in their retinal blood vessel segmentation works.

### 2.3. Pratt’S Figure of Merit (PFOM)

W. Pratt [41] introduced Pratt’s Figure of Merit (PFOM), which is a method used to provide a quantitative comparison between edge detection algorithms. PFOM is one of the popular assessment methods for edge detection [42]. PFOM is expressed mathematically with the formula in Equation (1).

(1) $$\mathrm{PFOM}=\frac{1}{N}\sum_{i=1}^{N_{A}}\frac{1}{1+\alpha d_{i}^{2}}$$

where $N=\max\{N_{I},N_{A}\}$, and $N_{I}$ and $N_{A}$ are the number of the ideal and actual edge map points, respectively. In this equation, α is a scaling constant, and $d_{i}$ is the separation distance of the actual edge point *i* normal to a line of ideal edge points. The rating factor is normalized so that PFOM = 1 is for a perfectly detected edge. The scaling factor may be adjusted to penalize edges that are localized but offset from the true position. Normalization by the maximum of the actual and ideal number of edge points ensures a penalty for smeared or fragmented edges. The PFOM is useful to consider errors such as missing valid edge points, failure to localize edge points, and classification of noise fluctuations as edge points.

## 3. Proposed Segmentation Approach

This section will be divided into four subsections. First, the dataset used in this work is presented in Section 3.1. Then, the overall flow of the proposed approach is presented in Section 3.2. Next, the edge detection method used in this work is presented in Section 3.3. After that, the toolbar parameters are presented in Section 3.4, and the graphical user interface (GUI) is presented in Section 3.5.

### 3.1. Dataset

**Figure 1:**
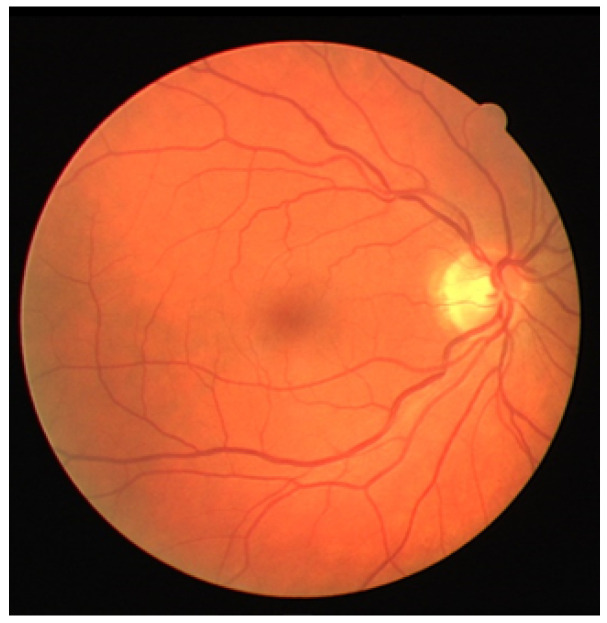
Example of a fundus image from DRIVE dataset.

The dataset used for testing, results, and evaluation is a publicly available dataset “DRIVE: Digital Retinal Images for Vessel Extraction” on the website “Grand Challenge” (https://drive.grand-challenge.org/, accessed on 15 May 2021).The DRIVE database has been established to enable comparative studies on the segmentation of blood vessels in retinal images. An example of a fully colored retinal fundus image from this dataset is shown in Figure 1.

### 3.2. Overall Flow of Program

**Figure 2:**
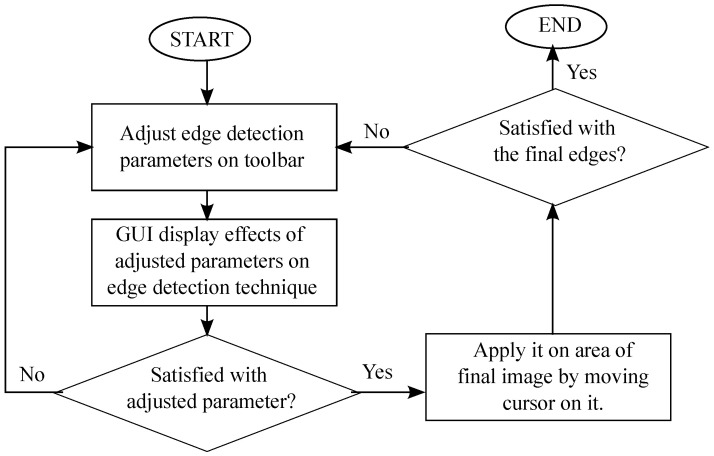
Flowchart of program.

Figure 2 shows the general flow of the program. First, the input image is read, and a modified version of Canny edge detection is applied to the image. Next, by using the designed GUI, the user can easily change the parameters of the edge detection method and observe how it affects the resulting image by using a toolbar from the GUI. The user can apply the edge detection with the selected parameters onto a part by clicking and moving the mouse around the desired part. Next, the user can readjust the parameters and repeat the process until the desired output image is produced.

### 3.3. Edge Detection Method

**Figure 3:**
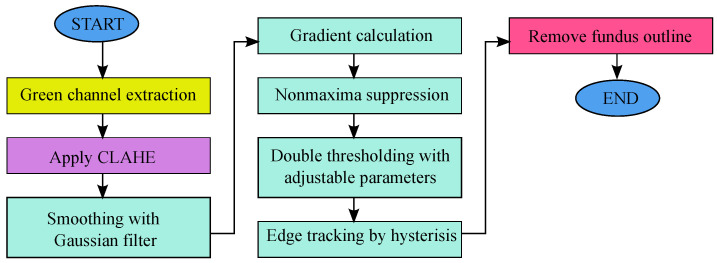
Flowchart of the modified edge detection.

The Canny edge detector with enhanced features is proposed in this paper to help in segmenting the vessel semi-automatically. The parameters of the detector are adjustable via the GUI interface. The modified edge detector consists of multiple stages, as shown in Figure 3. In this figure, the blocks from the original Canny edge detector are colored green, whereas other colors present the added features.

The proposed approach only works on the eight-bit-depth image. Both CLAHE and the Canny edge detector are only executed on a single channel. As the input to the system is a color retina fundus image, the first step in this method is to split the RGB color into three channels, which are the red channel (R), the green channel (G), and the blue channel (B). Then, the green channel is kept as the input image, and thus the 24-bit-depth color image has now become an eight-bit-depth image. In comparison to the grayscale channel converted from the RGB channels, the green channel has a better local contrast between the foreground and the background [43].

In some cases, the adjacent regions have similar intensity values. As a consequence, the edges between these regions are weak due to relatively low gradient values. Therefore, to increase the contrast for such regions, in this work, CLAHE is utilized on the green channel G of the image. CLAHE enhances the image by using the local cumulative distribution function (CDF) as its local mapping function. To avoid unnatural enhancement and noise amplification, CLAHE limits the contrast enhancement by trimming the histogram at a predefined clip limit before computing the CDF. This regulates the slope of CDF and, therefore, of the transformation function. The clip limit depends on the normalization of the histogram and hence on the size of the contextual region. The contrast in the uniform area is restricted to avoid intensifying the noise. If any histogram bin is exceeding the threshold value, those histograms are clipped and distributed uniformly to other bins before applying histogram equalization. The code for applying CLAHE is done using the OpenCV library. First, a CLAHE object is created with a clip limit of 2.0 and a default contextual region’s size of 15×15 pixels if no changes are made to the toolbar. Then, the CLAHE object is applied onto the green (G) channel of the fundus image.

The next step is by implementing the Canny edge detector. The first stage of this process is to reduce the noise level by filtering the image with a Gaussian filter. The image is convolved with a Gaussian filter using GaussianBlur(), which can be expressed by Equation (2):

(2) $$G\left(x,y\right)=\exp\left(-\frac{x^{2}+y^{2}}{2\sigma^{2}}\right)$$

where *x* and *y* are the spatial coordinates on the Gaussian filter, and σ is the predefined standard deviation. In this work, the Gaussian kernel size is set to be 5×5 pixels as the default if no changes are made to the toolbar. The Gaussian kernel standard deviation in the *x*-direction and *y*-direction is set as 1.4.

In the next stage, we compute the first derivative in the vertical direction ($g_{y}$) and horizontal direction ($g_{x}$) by using the Sobel kernels in both vertical and horizontal directions. Subsequently, the information about the gradient magnitude *M* and angle θ is determined by changing the Cartesian coordinates to polar coordinates, as given by Equations (3) and (4):

(3) $$\begin{array}{c} M\left(x,y\right)=\sqrt{g_{x}^{2}+g_{y}^{2}} \end{array}$$

(4) $$\begin{array}{c} \theta \left(x,y\right)=\tan^{-1}\left(\frac{g_{y}}{g_{x}}\right) \end{array}$$

Then, with the aim to make the wide ridges around the local maxima to become thinner, a non-maxima suppression is utilized to the gradient magnitude. A loop through every pixel of the image is used to produce a non-maxima suppressed image. Four discrete orientations of the edge normal (gradient vector) are defined, which are horizontal, vertical, top right ($45^{\circ }$), and top left ($-45^{\circ }$). In each loop, if the magnitude M(x,y) of that pixel is lower than one or both neighboring points along the orientations, it will be set to zero.

After that, double thresholding is exploited to lower false edge points. All pixels are grouped into three types, which are strong, weak, and irrelevant, based on two thresholds adjustable in the GUI. Next, hysteresis is done to track edges. A loop is run for each pixel, where the weak pixels will be transformed into strong pixels if and only if they have one strong neighboring pixel. If not, the weak pixel will be irrelevant.

In the final step, the fundus outline is removed. First, by thresholding, the extracted green channel using a simple threshold to separate black colors (i.e., the background of the fundus image) with other colors (i.e., the retinal itself), a binary mask of the retinal is created. Next, the binary mask of the background is eroded to expand it slightly into the retinal outline. Then, a bitwise AND operation is utilized on the Canny image and the binary mask of the background. By using this approach, the retinal outline will be removed by the mask.

In some cases, CLAHE may fail to increase the contrast in some areas of the image. This condition may affect the edges detected by the default Canny edge detector, where edges between areas with similar intensity may not be detected successfully. However, the proposed semi-automatic method in this paper can overcome this problem by using three approaches:

1. Interactively changing the size of the filters used for CLAHE and Gaussian smoothing. By changing the parameters of the filter, it may help us to get a better-contrasted image.
2. Changing the threshold values (i.e., low or high threshold values) for the Canny edge detector. As the threshold value can be changed interactively, we can set the threshold values locally. Regions with strong edges can utilize high threshold values, whereas regions with weak edges can utilize relatively lower threshold values.
3. If both approaches mentioned above fail to produce the intended weak edges, our approach allows the user to switch to a manual mode. Using this mode, the user can define the edges themselves. However, we believe that the quantity of these weak edges is relatively low compared to the strong edges. Thus, in general, the partition where the edges need to be segmented is low in this approach.

### 3.4. Toolbar Parameters

The parameters of this edge detection method can be easily adjusted using a sliding bar on the toolbox in the GUI designed. The parameters that can be adjusted are the strong threshold, weak threshold, Gaussian blur kernel, and the tile grid size of CLAHE.

After the procedure of non-maximum suppression, the residual edge pixels will provide a more truthful representation of real edges in an image. However, some edge pixels that are remained are actually caused by noise and color variation. Therefore, it is crucial to filter out edge pixels with a weak gradient value and preserve edge pixels with a high gradient value to account for these spurious responses. This is achieved by choosing suitable high and low threshold values. If an edge pixel’s gradient value is greater than the high threshold value, it is denoted as a strong edge pixel. If an edge pixel’s gradient value is lesser than the high threshold value and higher than the low threshold value, it is denoted as a weak edge pixel. If an edge pixel’s gradient value is smaller than the low threshold value, it will be suppressed. The two threshold values are empirically determined, and their definition will be subject to the content of a given input image.

The Gaussian blur kernel’s size affects the noise of the image. In this work, the contextual region or the tile gride size of CLAHE is set to be triple the size of the Gaussian kernel and is changed whenever the size of the Gaussian kernel is adjusted. The CLAHE tile grid size should be large enough to avoid intensifying noise that could be suppressed by the Gaussian kernel but should be small enough to enhance the edges.

### 3.5. Graphical User Interface (GUI)

For designing the GUI, Python is used with some open-source libraries such as OpenCV, NumPy, Tkinter, and Pyinstaller. An executable file of the GUI is created using the Pyinstaller library.

When the GUI is started, the user will be asked to select the fundus image to be segmented. After the image file is read, the input fundus image and the image after edge detection is applied will be displayed. The toolbar used to adjust the parameters will also be displayed. This will allow the user to easily see the effects of the adjusted parameter on the edge detection while adjusting it on the toolbar. A sliding bar is used for adjusting the parameters in the toolbar due to its simplicity and convenience.

When the cursor is pressed and moved along a vessel, automatic edge detection will be done around the vessel. The purpose of this GUI design is to be direct and allow users to easily segment the vessels without much effort. The color used to draw the edges can be selected as a method to mark specific edges or categorizing certain edges together. The parameters for edge detection can be adjusted using the toolbar during the segmentation of different vessels. While adjusting the parameters on the toolbar, the GUI will display the effects on the edge detection so that the ideal parameter will be chosen. The GUI also includes basic tools such as manually drawing the lines, erasing the lines, and labeling the blood vessels. The GUI tools can be controlled by pressing keys on the keyboard, which are shown in Table 1.

## 4. Results and Discussions

This section is divided into three subsections. Section 4.1 will present the GUI design. Section 4.2 will present the GUI toolbar. Section 4.3 is for edge detection technique results and evaluation Section 4.3.

### 4.1. Graphical User Interface Design

**Figure 4:**
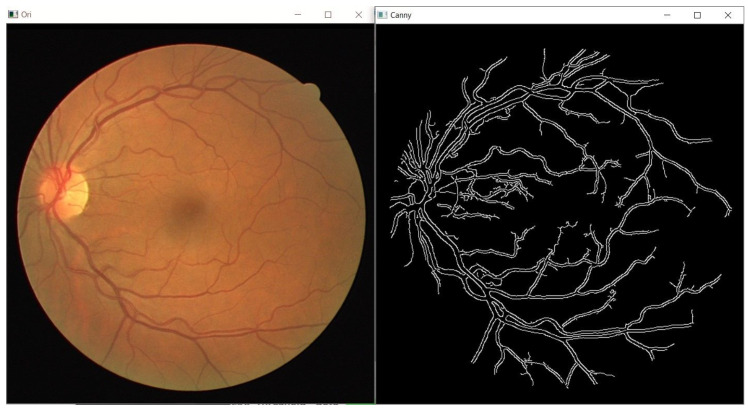
Image of input retinal fundus image and its image after edge detection is applied.

**Figure 5:**
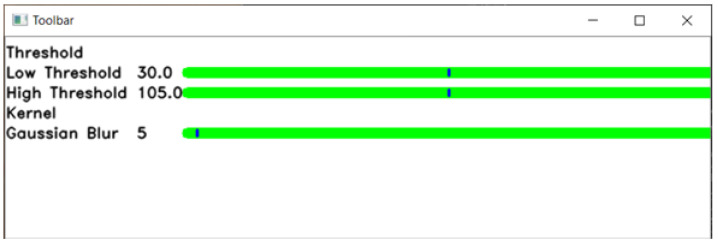
Image of toolbar.

The GUI starts running after the user runs the executable file created for it. When the GUI is started, the program first asks the user to select the input image, which is the fundus image in the computer. After the input fundus image is selected by the user, the fundus image and its image after edge detection is applied as displayed in Figure 4. The toolbar used to adjust the parameters will also be displayed, as shown in Figure 5.

**Figure 6:**
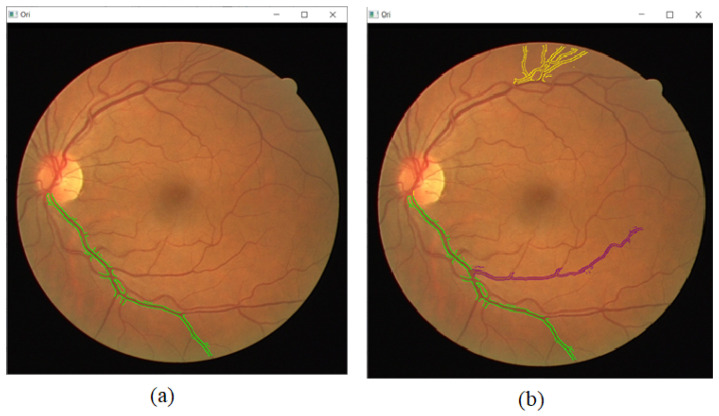
(**a**) Automatic edge detection is done on a vessel when a cursor is moved along it. (**b**) Multiple colors are used to categorize and differentiate the vessels for analysis.

During segmentation, when the cursor is pressed with a left-click and moved along a vessel, automatic edge detection will be done around the vessel, as shown in Figure 6a. The method and parameters of automatic edge detection can be changed accordingly. The color used to draw the edges can be selected as a method to mark specific edges or categorizing certain edges together, which is shown in Figure 6b. Note that the parameters for edge detection are also adjusted using the toolbar during segmentation of the three vessels on Figure 6b. While adjusting the parameters on the toolbar, the GUI will display the effects on the edge detection so that the ideal parameter will be chosen. Lower thresholds are set for the yellow edges, as the blood vessel it highlights is much smaller and requires lower thresholds to be detected. The thresholds are set as more average, which is the default toolbar value when segmenting the blue edges. The thresholds are set much higher when segmenting the green edge because the blood vessel is much bigger and can be more easily detected. The purpose of the higher thresholds is to make the edges smoother. Besides that, the colored edges can make it much easier to point to specific vessels during discussion or presentation. The Gaussian kernel blur is also adjusted accordingly to remove noises. The GUI also includes basic tools such as manually drawing the lines, erasing the lines, and labeling the blood vessels.

**Figure 7:**
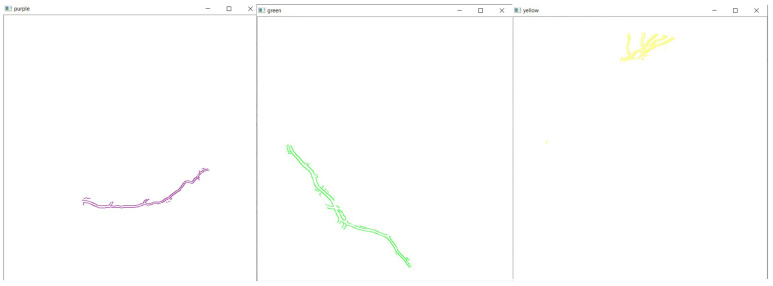
Segmentation of detected edges are displayed separately.

**Figure 8:**
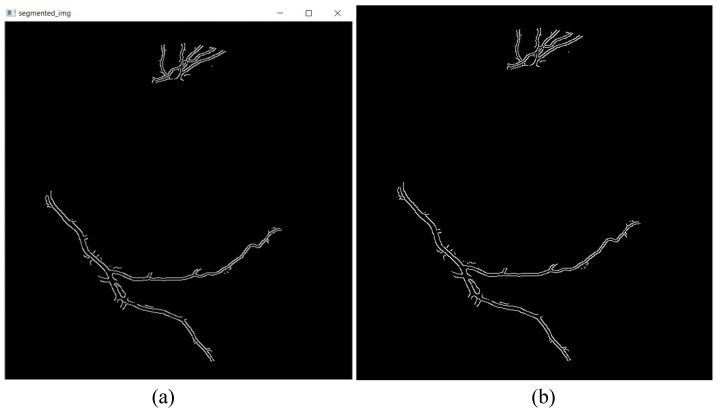
(**a**) Window displaying combination of all segmented parts. (**b**) Image created and saved as “segmented_img.jpg”.

Finally, the detected edges are segmented out and displayed separately to be analyzed individually, as shown in Figure 7. A combination of all segmented parts will be displayed, as shown in Figure 8. The image is also created as a jpg file, named “segmented_img.jpg”, and stored in the same folder location as the GUI executable file.

### 4.2. Graphical User Interface Toolbar

**Figure 9:**
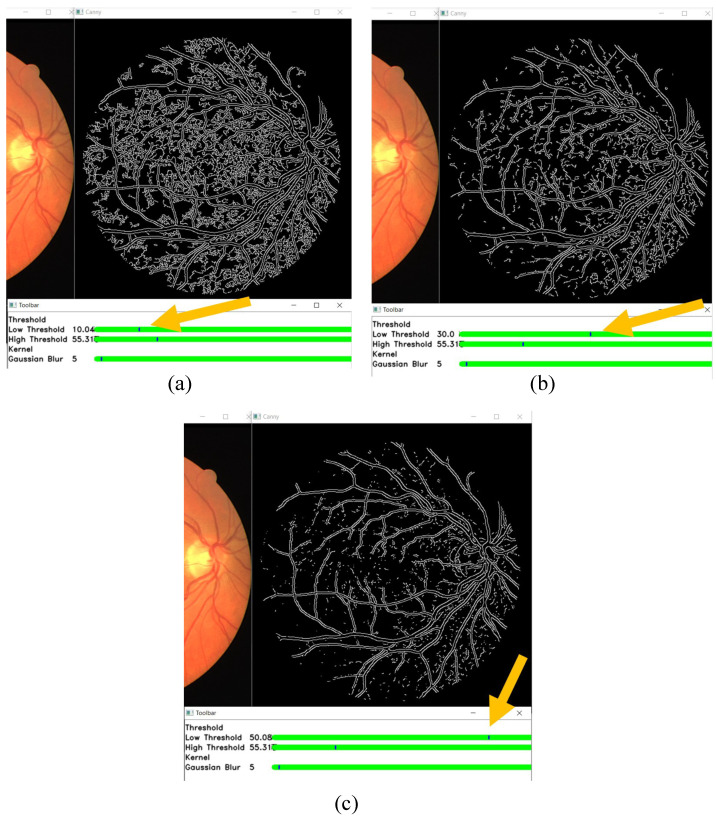
High threshold at 55, and low threshold at (**a**) 10 (the obtained PFOM value is 0.4739), (**b**) 30 (the obtained PFOM value is 0.5448), and (**c**) 50 (the obtained PFOM value is 0.5830).

**Figure 10:**
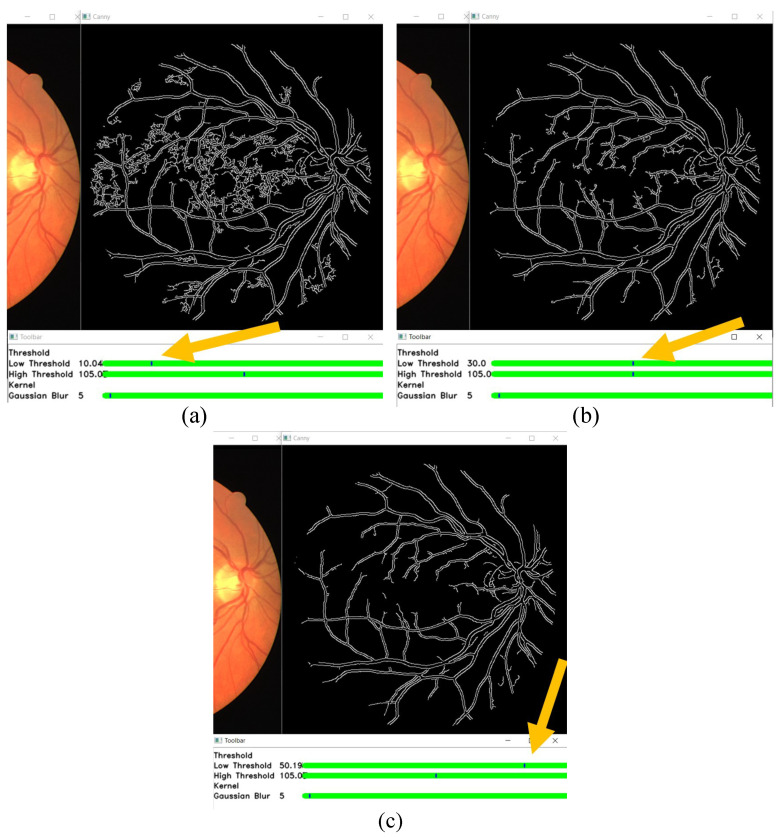
High threshold at 105, and low threshold at (**a**) 10 (the obtained PFOM value is 0.5886), (**b**) 30 (the obtained PFOM value is 0.600), and (**c**) 50 (the obtained PFOM value is 0.5511).

**Figure 11:**
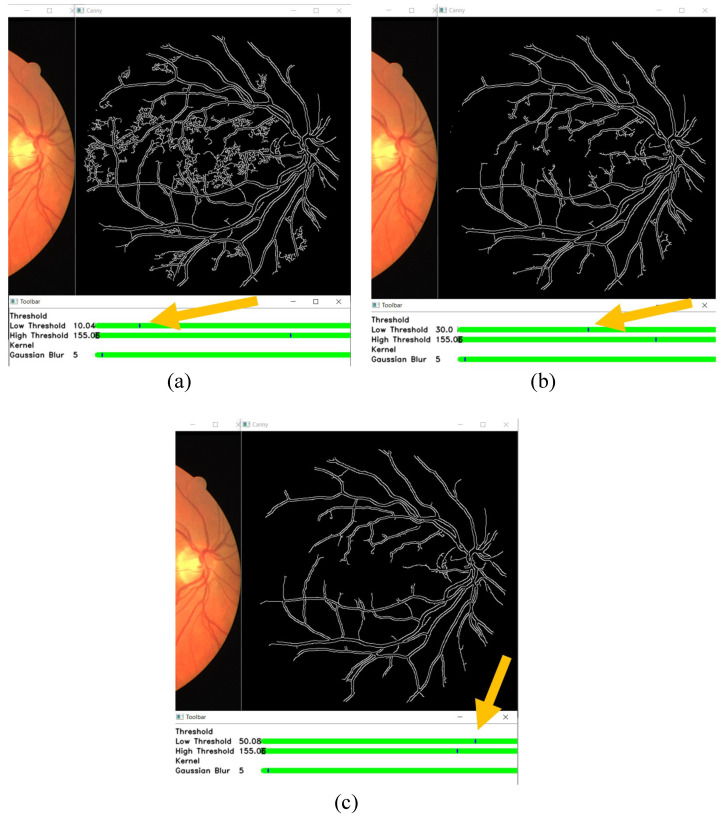
High threshold at 155, and low threshold at (**a**) 10 (the obtained PFOM value is 0.5941), (**b**) 30 (the obtained PFOM value is 0.5532), and (**c**) 50 (the obtained PFOM value is 0.5025).

In this section, the results of changing the edge detection parameters using the GUI toolbar are shown, which are the low threshold, high threshold, Gaussian kernel, and CLAHE tile size. The effects of changing the parameter on the toolbar are recorded in the following figures. Figure 9, Figure 10 and Figure 11 show a visual comparison of the effects of changing the threshold parameters on the toolbar, together with the measured PFOM values. In these figures, the yellow arrows indicate the location where we change the trackbar. Figure 1 is used as the input fundus image used by Figure 9, Figure 10 and Figure 11. The figures show that increasing the low threshold will reduce the number of noises detected, while decreasing the low threshold will increase the number of edges detected. Meanwhile, increasing the high threshold can reduce the noises around the edges detected, while decreasing the high threshold can strengthen the edges detected.

In terms of the observed PFOM values, in Figure 9, which is when the high threshold is set to 55, it is observed that when we increase the low threshold value, the value of PFOM is increased. The reason behind this observation is that, by increasing the low threshold value, more unwanted edges will be removed. For the case in Figure 10, in which the high threshold is 105, when we increase the low threshold from 10 to 30, the PFOM value is increased. This is because the false edges have been reduced. However, when we increased the low threshold from 30 to 50, the PFOM value decreased. This is because the detector starts to remove useful edges. The same reason is also applied to the case of Figure 11, where the PFOM values decreased when the low threshold value increased. This observation also shows the Canny edge detector’s difficulty in detecting good edges by using only one set of global threshold values. This becomes one of the reasons why we allow those threshold values to be set interactively so that suitable threshold settings can be applied locally.

**Figure 12:**
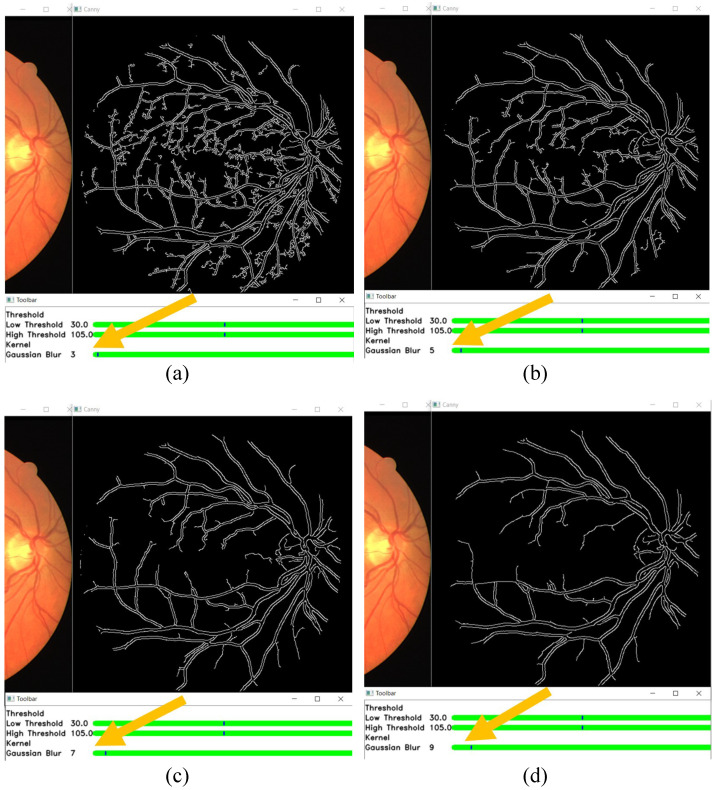
Edges detected by using a Gaussian filter of size (**a**) 3×3 pixels, (**b**) 5×5 pixels, (**c**) 7×7 pixels, and (**d**) 9×9 pixels.

Figure 12 shows the effect of changing the size of the kernel in the GUI toolbar. The yellow arrows in this figure show the location of the changes of the settings that are done on the trackbar. A visual comparison of the effects of Gaussian kernel size 3, 5, 7, and 9 can be made. Figure 1 is used as the input fundus image. The other parameters are set as the default values, which are the low threshold of 30 and the high threshold of 105. From Figure 12, it is shown that a smaller Gaussian kernel size can detect more edges. However, it is also going to falsely detect noises as edges. A bigger Gaussian kernel size can suppress more noise but also falsely suppresses the smaller veins as noises instead.

### 4.3. Edge Detection Technique Results and Evaluation

**Figure 13:**
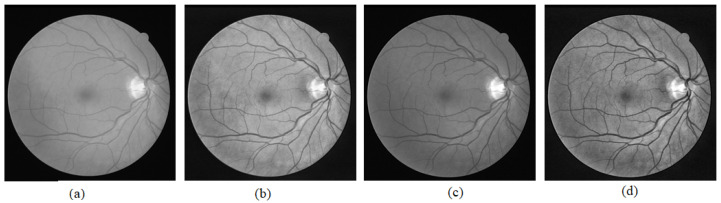
Green channel extraction and CLAHE on Photo 1. (**a**) Grayscale image. (**b**) CLAHE on grayscale image. (**c**) Extracted green channel. (**d**) CLAHE on extracted green channel.

**Figure 14:**
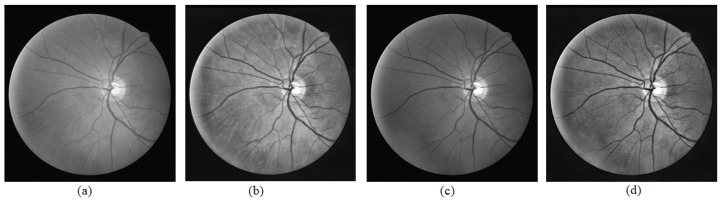
Green channel extraction and CLAHE on Photo 2. (**a**) Grayscale image. (**b**) CLAHE on grayscale image. (**c**) Extracted green channel. (**d**) CLAHE on extracted green channel.

Figure 13 and Figure 14 show visual comparisons of the fundus images from the effect of extracting green channel and applying CLAHE, which are techniques used in the GUI. Figure 13 and Figure 14 show the fundus images after converting them to grayscale, the fundus images after applying CLAHE on the grayscale image, the fundus images after extracting the green channel from the RGB channel, and the fundus images after applying CLAHE onto the green channel. The input images used are taken from the DRIVE dataset. From the visual comparison, extracting the green channel and applying CLAHE make the blood vessels clearer in the fundus photo to improve edge detection.

Table 2 shows the highest possible PFOM value of 10 images after running the Canny edge detector on the grayscale image, the extracted green channel, and after applying CLAHE on the extracted green channel. The input photos and their ground truth image used are taken from the DRIVE dataset. A comparison can be made between the 3 PFOM values (calculated using their ground truth provided in the DRIVE dataset) to see the effects of extracting the green channel from the fundus image and applying CLAHE on the extracted green channel.

From Table 2, the PFOM value is higher when running the Canny edge detector on the extracted green channel from the fundus photo compared to running the Canny edge detector on the grayscale image converted from the fundus photo. The highest possible PFOM value also increases when CLAHE is applied to the extracted green channel. This shows that extracting the green channel and applying CLAHE can raise the edge detector’s potential to detect edges accurately. Besides that, from Table 2, the ideal low threshold and the ideal high threshold for the extracted green channel are higher than the grayscale image. This is because the extracted green channel has the maximum local contrast between the background and the foreground. Increasing the local contrast would make the blood vessel edges easier to be detected, which increases the ideal low and high threshold, resulting in suppressing more noises. Similarly, applying CLAHE increases the ideal low threshold and ideal high threshold.

**Figure 15:**
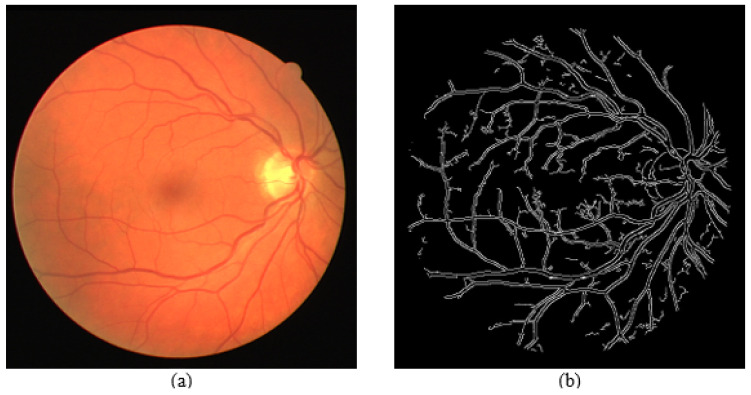

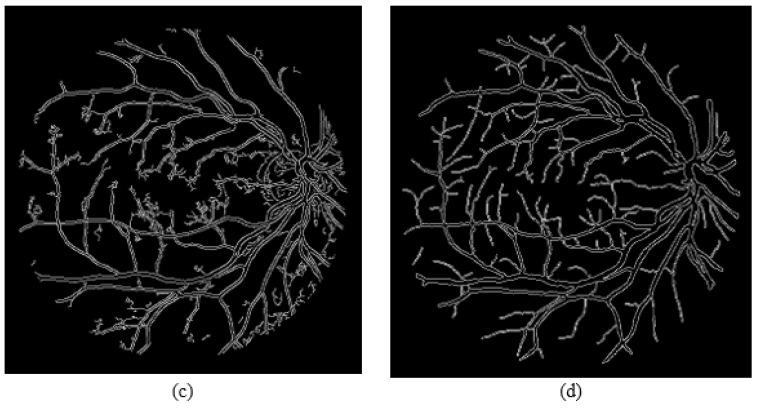
Comparison of segmented images for Photo 1. (**a**) Input image. (**b**) Segmented image using the proposed approach (GUI based). (**c**) Segmented image using Canny detector with ideal parameters. (**d**) Ground truth image from DRIVE dataset.

**Figure 16:**
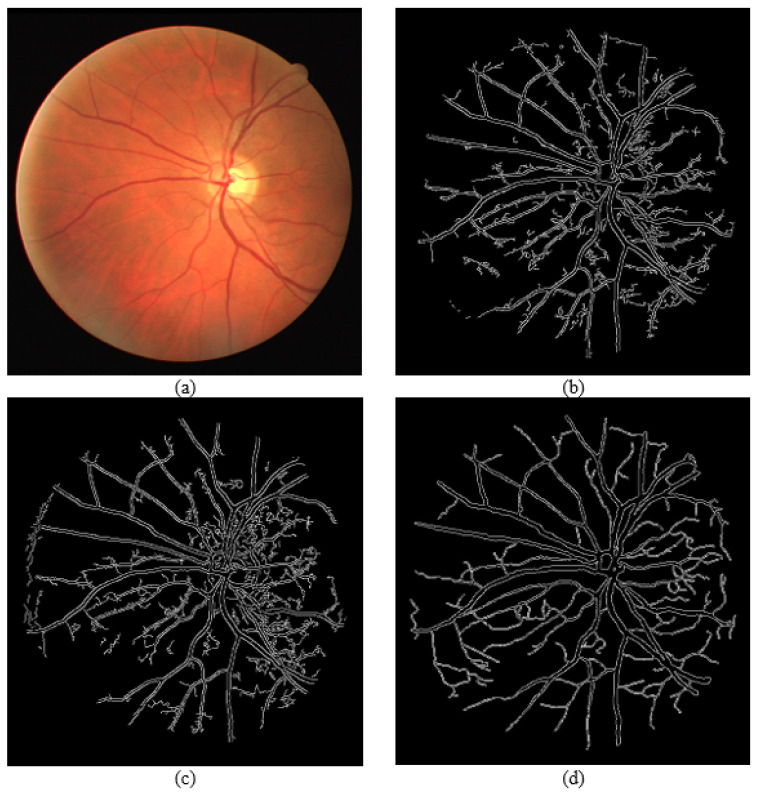
Comparison of segmented images for Photo 2. (**a**) Input image. (**b**) Segmented image using the proposed approach (GUI based). (**c**) Segmented image using Canny detector with ideal parameters. (**d**) Ground truth image from DRIVE dataset.

**Figure 17:**
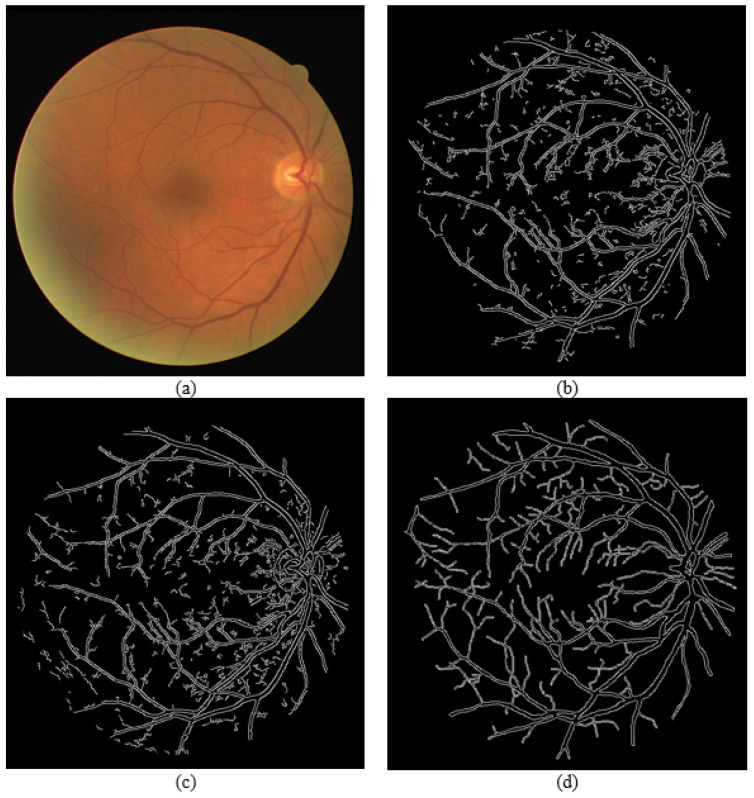
Comparison of segmented images for Photo 3. (**a**) Input image. (**b**) Segmented image using the proposed approach (GUI based). (**c**) Segmented image using Canny detector with ideal parameters. (**d**) Ground truth image from DRIVE dataset.

Figure 15, Figure 16 and Figure 17 show results from segmenting three different fundus photos using the GUI. The input fundus photos are taken from the DRIVE dataset. A visual comparison is made with the segmented image produced from the GUI, the segmented image using default Canny detector with ideal thresholds, with retinal outline manually removed, and the golden segmented image from the DRIVE dataset.

From the comparison of the figures shown, the segmented images using the GUI are mostly visually similar to the segmented images using the default Canny edge detector. However, a closer inspection would show that the segmented images using the GUI have less noise falsely detected and is able to detect more true edges. The high similarity is due to the edge detection technique incorporated by the GUI contains mainly the Canny edge detector itself. However, the added features into the edge detector, along with the GUI function of applying a different threshold on different areas is able to improve the edge detection of the fundus image.

Table 3 shows the PFOM value from the image segmented using the GUI with comparison to the default Canny edge detector with the ideal thresholds. From the comparison, the segmented images using the GUI have a higher PFOM value than the default Canny edge detector.

**Figure 18:**
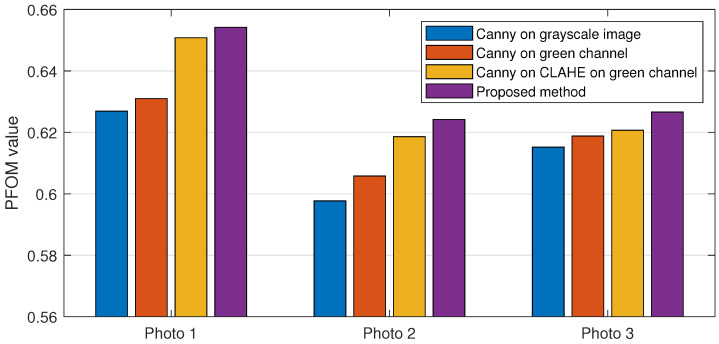
Comparison of PFOM values of photos 1 to 3 from DRIVE dataset with different segmentation methods and different processing on input image.

A comparison of the highest PFOM values of the three images and the PFOM value of segmented images using the GUI is shown in a bar chart in Figure 18. This figure clearly shows that the proposed segmentation approach (i.e., GUI-based) produces the highest PFOM value for these three test images.

**Figure 19:**
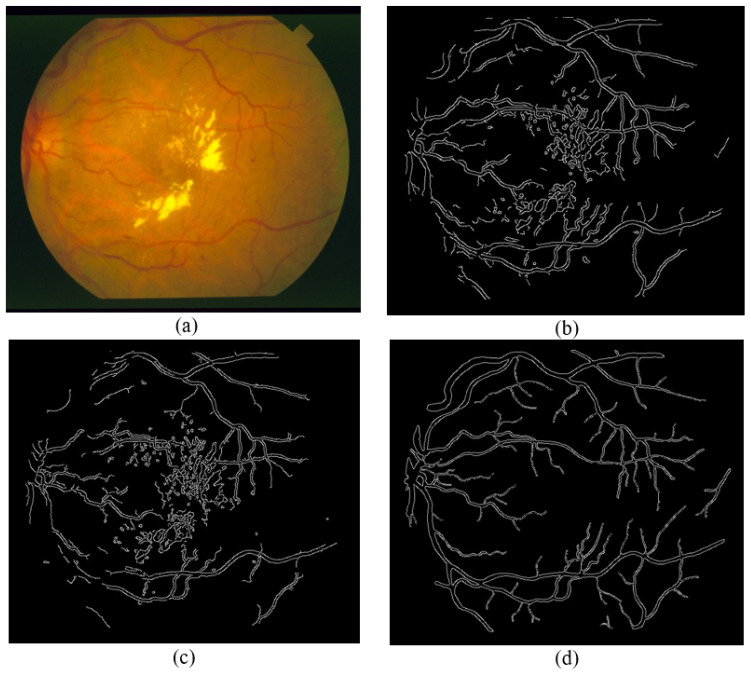
Comparison of segmented images for image 0001 from STARE dataset. (**a**) Input image. (**b**) Segmented image using the proposed approach (GUI based) (the obtained PFOM value is 0.5208). (**c**) Segmented image using Canny detector with ideal parameters (i.e., low threshold is set at 10, and high threshold is set at 32) (the obtained PFOM value is 0.4346). (**d**) Ground truth image from STARE dataset.

In order to show that the proposed method can work in a wide range of input retina fundus images, we have also tested the performance of the proposed semi-automatic segmentation method on other dataset. Figure 19 shows the segmentation results of image 0001 from STARE dataset [44,45]. It is shown that the proposed method works better than the Canny edge detector with the ideal thresholds.

**Figure 20:**
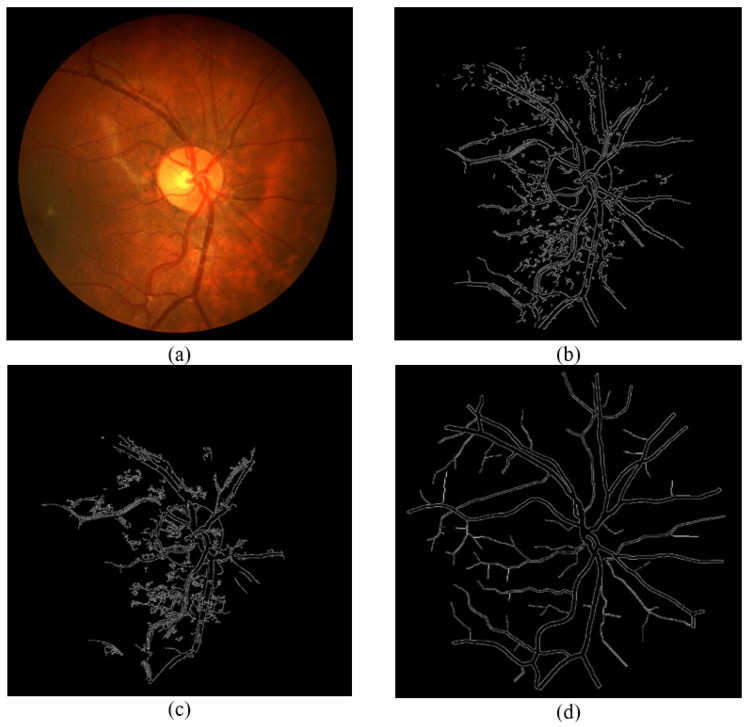
Comparison of segmented images for Image_01R.jpg from the CHASE_DB1 dataset. (**a**) Input image. (**b**) Segmented image using the proposed approach (GUI based) (the obtained PFOM value is 0.5208). (**c**) Segmented image using Canny detector with ideal parameters (i.e., low threshold is set at 6, and high threshold is set at 60) (the obtained PFOM value is 0.3664). (**d**) Ground truth image from the CHASE_DB1 dataset.

Figure 20 presents the results of the segmentation of image Image_01R.jpg from the CHASE_DB1 dataset [46]. Again, the proposed method produces better output as compared with the Canny edge detector with the ideal thresholds. The PFOM value from the proposed method (Figure 20b) is also higher than the one obtained from the Canny edge detector (Figure 20c).

## 5. Conclusions

Retinal vessel segmentation is an important technique to inspect arteries and veins in the retina. In this paper, a vessel segmentation Graphical User Interface (GUI) is proposed, where semi-automated segmentation of specific vessels can be done by simply moving the cursor across a particular vessel. Edge detection techniques (modified version of Canny) will be applied on the vessel selected, and the program will draw out the vessel edges. The GUI also functions to apply edge detection with different parameters on different areas of the same image to fully optimize the edge detection, with the help of a toolbar to adjust the parameters. The added features are extracting the green channel, applying CLAHE, and removing the retinal outline. Based on the results presented in this paper, the proposed GUI method proves to be better than the traditional edge detector. The proposed GUI is also providing the automatic convenience of the edge detector compared to the manual segmentation method. The added features of the modified edge detection technique are also shown to improve the Canny detector.

The proposed GUI method still contains limitations as the modified edge detector still cannot completely accurately detected edges or completely remove noises. For future research, the edge detection technique can be further improved to detect edges accurately. Besides that, a better noise filter can be implemented in future research to reduce noise further. Besides that, more features can be added to the GUI program itself to allow more convenience, functions, and flexibility. The potential of the GUI itself is infinite and only limited by the creativity of the designer itself. For future research, more work can be done to add to the GUI’s features.

## Figures and Tables

**Table 1:** Functions of keys.

Key	Function
q	End program
b	Detect edges with blue-colored lines
g	Detect edges with green-colored lines
p	Detect edges with purple-colored lines
y	Detect edges with yellow-colored lines
w	Remove detected edges line drawn
m	Change to manual segmentation mode
a	Change to automated segmentation mode
d	Produce output images

**Table 2:** Highest PFOM value for grayscale image, extracted green channel of image, and CLAHE on extracted green channel of image.

Fundus Image	Highest PFOM Value (Ideal Low Threshold, Ideal High Threshold)		
	Grayscale Image	Extracted Green Channel	Apply CLAHE on Extracted Green Channel
1	0.6269 (4/5, 27)	0.6310 (8/9, 35)	0.6508 (30/32, 76)
2	0.5977 (6/7, 29)	0.6058 (10/11, 31)	0.6186 (28/29, 64)
3	0.6152 (8/9, 21)	0.6188 (8/9, 25)	0.6207 (26/27, 57)
4	0.6015 (8/9, 25)	0.6097 (14/15, 23)	0.6110 (22/23, 75)
5	0.6132 (10/11, 21)	0.6180 (10/11, 23)	0.6230 (24/25, 63)
6	0.6285 (14/15, 23)	0.6339 (12/13, 27)	0.6362 (36/37, 67)
7	0.6151 (8/9, 31)	0.6210 (8/9, 39)	0.6223 (26/27, 90)
8	0.6401 (8/9, 27)	0.6455 (14/15, 25)	0.6478 (30/31, 70)
9	0.6110 (14/15, 25)	0.6210 (8/9, 39)	0.6325 (26/27, 100)
10	0.6475 (10/11, 25)	0.6479 (10/11, 29)	0.6516 (30/31, 72)

**Table 3:** Comparison of PFOM for segmented images.

Fundus Image	Pratt’s Figure of Merit (PFOM) Value	
	Segmented Image Using the Default Canny Detector (Ideal Low Threshold, Ideal High Threshold)	Segmented Image Using the Proposed Method (GUI Based Method)
Photo 1	0.6269 (5, 27)	0.6542
Photo 2	0.5977 (7, 29)	0.6242
Photo 3	0.6152 (9, 21)	0.6266

## Data Availability

The three datasets used in this work are publicly available. They are (i) ‘’DRIVE: Digital Retinal Images for Vessel Extraction” in the website “Grand Challenge” (https://drive.grand-challenge.org, accessed on 15 May 2021), (ii) “STARE‘’ in website “Structured Analysis of the Retina” (https://cecas.clemson.edu/~ahoover/stare/, accessed on 11 September 2021), and (iii) “CHASE_DB1” in website “Retinal Image Database” (https://blogs.kingston.uc.uk/retinal/chasedb1, accessed on 10 September 2021).
